# Influence of Ag on the Properties of Ca_0.9_Yb_0.1_MnO_3_ Sintered Ceramics

**DOI:** 10.3390/ma11122503

**Published:** 2018-12-09

**Authors:** Andrés Sotelo, Miguel A. Torres, María A. Madre, Juan C. Diez

**Affiliations:** Instituto de Ciencia de Materiales de Aragón (CSIC-Universidad de Zaragoza), C/María de Luna, 3, 50018 Zaragoza, Spain; matorres@unizar.es (M.A.T.); amadre@unizar.es (M.A.M.); monux@unizar.es (J.C.D.)

**Keywords:** thermoelectricity, manganese oxide, silver, sintering, power factor

## Abstract

In this study, Ca_0.9_Yb_0.1_MnO_3_ + x wt.% Ag (with x = 0, 1, 3, 5, and 10) thermoelectric materials were prepared via the classical ceramic method. In spite of the very high sintering temperature (1300 °C), no significant Ag losses were observed following this process. Moreover, Ag addition enhanced cation mobility during sintering due to the formation of a liquid phase. Microstructurally, it was found that Ag decreases porosity; this was confirmed by density measurements. Ag was also found to promote the formation of a Ca_2_Mn_2_O_5_ secondary phase. Despite the presence of this secondary phase, samples with Ag displayed lower electrical resistivity than Ag-free ones, without a drastic decrease in the absolute Seebeck coefficient. The highest thermoelectric performances, which were determined by power factor, were obtained in 1 wt.% Ag samples. These maximum values are slightly higher than the best of those reported in the literature for sintered materials with similar compositions, with the additional advantage of their being obtained using a much shorter sintering procedure.

## 1. Introduction

Thermoelectric (TE) devices can transform heat to electrical energy without using moving components. Consequently, they can increase the efficiency of energy-transforming systems [1] and decrease associated CO_2_ emissions, thereby playing an important role in the fight against global warming. These TE devices are built with p- and n-type TE materials connected electrically in series and thermally in parallel. At present, commercial devices are built with metal-based materials [2,3], with Bi-Te-based ones used most often due to their high TE performance. Unfortunately, their easy oxidation and/or evaporation at moderately high temperatures and in air drastically limits their use under these conditions [1]. On the other hand, in spite of their currently lower TE performance, oxide materials display much higher thermal and chemical stability in oxidative environments. They also have a higher relative abundance in the earth’s crust, are cheaper, and are less toxic. The main objective concerning these oxides is to enhance their TE figure-of-merit (ZT). This is defined as TS^2^/ρκ (S^2^/ρ is also called the power factor (PF)), where T, S, ρ, and κ are absolute temperature, Seebeck coefficient, electrical resistivity, and thermal conductivity, respectively [4].

These TE oxide materials belong to the family of transition metal oxide (TMO), due to the presence of transition metals within their composition. In these TMOs, p- and n-type conducting materials can be found, with the former consisting of either Bi–Ca–Co–O or Ca–Co–O [5,6,7], and the latter being either Ca–Mn–O or Sr–Ti–O [8,9,10]. Among these, p-type materials have been studied the most and usually show better TE properties than n-type materials [11,12,13]. Consequently, it is necessary to enhance the typical performance of the latter to raise the efficiency of thermoelectric modules. For this reason, MnO-based n-type materials possess a crucial advantage over those which are TiO-based, due to the possibility of producing the former without the need of reducing conditions during sintering [14,15,16,17]. However, pristine MnO compounds exhibit very poor electrical properties [18] due to their low carrier concentration, which can be enhanced by partially substituting calcium with rare earth cations [19]. Among these rare earth cations, one of the most effective, with regard to electrical properties, is Yb^3+^ [9,20,21,22].

Another way to enhance electrical properties in ceramic systems is to use metallic additions when both materials do not react under sintering conditions. One of the most useful metals for this purpose is Ag, which, besides enhancing electrical properties [23,24], is very good at improving mechanical properties [25,26,27]. Furthermore, even if Ag is molten at CaMnO_3_ sintering temperatures, no Ag losses are expected, as demonstrated in previous research [26].

The objective of this work was to improve the performance of Ca_0.9_Yb_0.1_MnO_3_/Ag composites via a short sintering process. This process can be performed by taking advantage of the beneficial effect of using a liquid phase, which allows faster cation diffusion at sintering temperatures. The modifications in structure and microstructure, induced by Ag addition, were studied and related to electrical properties, using Ag-free samples as a reference.

## 2. Materials and Methods 

Ca_0.9_Yb_0.1_MnO_3_ + x wt.% Ag composites (x = 0, 1, 3, 5, and 10) were prepared using CaCO_3_ (≥ 99%, Aldrich, St. Louis, MO, USA), Mn_2_O_3_ (99%, Aldrich), Yb_2_O_3_ (99.9%, Aldrich), and Ag (≥ 99.9%, Aldrich) commercial powders. They were mixed in stoichiometric proportions and ball milled in water media in an agate ball mill for 30 min. The resulting slurries were dried using infrared radiation, followed by calcination at 900 °C for 12 h to decompose CaCO_3_. The powders were subsequently cold-uniaxially pressed at 275 MPa into pellets which were sintered at 1300 °C for 90 min. Following this, a final furnace cooling took place.

Identification of phases was made via powder X-ray diffraction (XRD), without any correction and with 2θ, from 10–70° using a Rigaku D/max-B X-ray powder diffractometer (copper target X-ray tube, 40 kV, 30 mA, CuKα radiation). Archimedes’ method was used for several samples of each composition to determine density values, using theoretical densities of 4.99 g/cm^3^ for Ca_0.9_Yb_0.1_MnO_3_ [20], and 10.5 g/cm^3^ for Ag [28]. Microstructures of the different samples were observed on transversal fractured and longitudinal surfaces in a field emission scanning electron microscope (FESEM, Zeiss Merlin, Oberkochen, Germany). Qualitative composition of the different phases was illustrated by an energy dispersive spectrometry (EDS) device.

Electrical resistivity and the Seebeck coefficient were determined in a LSR-3 system (Linseis GmbH, Selb, Germany) between room temperature and 800 °C, under a He atmosphere, in steady state mode. These data were used to evaluate TE performance through PF values.

## 3. Results and Discussion

**Figure 1:**
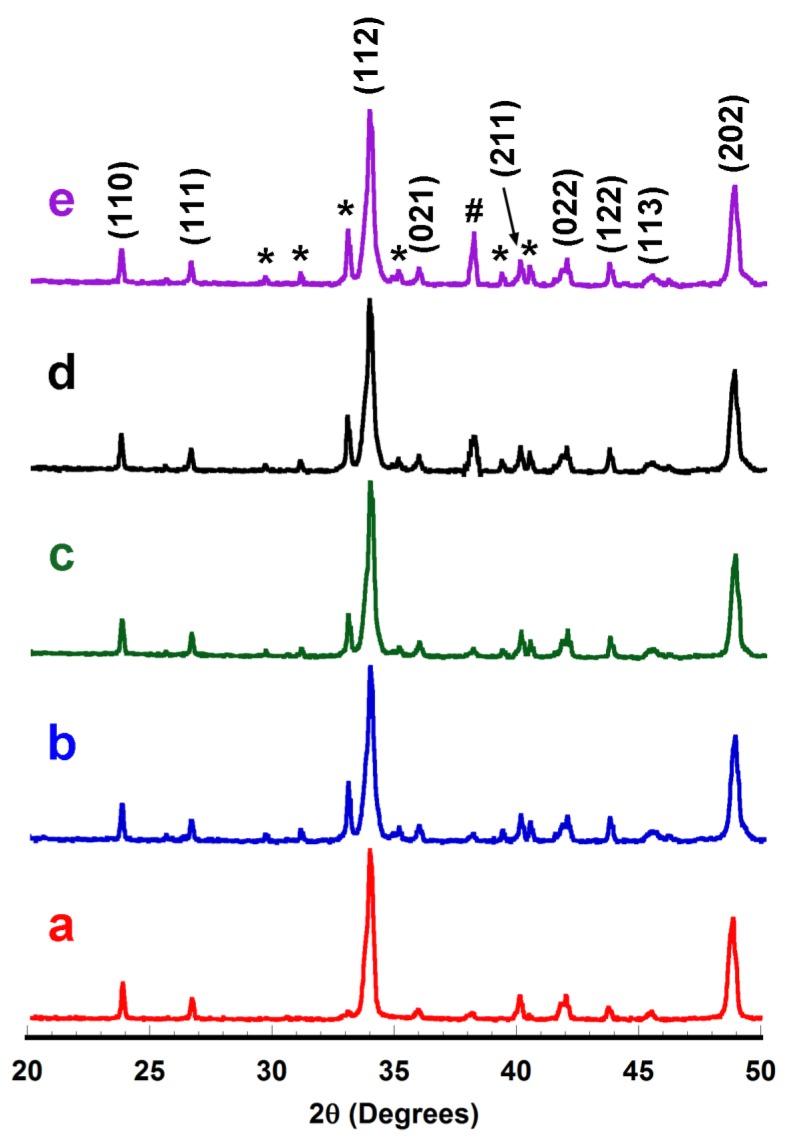
Powder X-ray diffraction (XRD) patterns, given in log scale on the y-axis, of Ca_0.9_Yb_0.1_MnO_3_ + x wt.% Ag samples after sintering for x = (**a**) 0; (**b**) 1; (**c**) 3; (**d**) 5; and (**e**) 10. Diffraction planes identify the peaks of the Ca_0.9_Yb_0.1_MnO_3_ phase, while * and # show those corresponding to Ca_2_Mn_2_O_5_ and metallic Ag, respectively.

Powder XRD patterns performed on sintered materials are displayed in Figure 1.

In Figure 1, it can be observed that Ca_0.9_Yb_0.1_MnO_3_ is the major phase (indicated by its diffraction planes) in all samples, independent of Ag addition, with a perovskite Pnma space group [29]. Additionally, the peaks identified by * and # indicate the presence of Yb-free Ca_2_Mn_2_O_5_ [30] and metallic Ag [31], respectively. The main difference observed between the samples is the formation of a Ca_2_Mn_2_O_5_ secondary phase when Ag is added to the samples. Furthermore, the amount of this secondary phase grew when Ag addition was increased. Consequently, it seems that the liquid phase (metallic Ag) produces a less formation of the thermoelectric Ca_0.9_Yb_0.1_MnO_3_ phase. This lower level of the thermoelectric phase can be linked to a lower oxygenation of the bulk sample, as has been reported for different systems [32,33], leading to the formation of a less oxygenated Ca_2_Mn_2_O_5_ phase. On the other hand, no shift in the XRD peaks was observed, indicating that Ag does not occupy any lattice site, and is maintained as metallic Ag.

**Figure 2:**
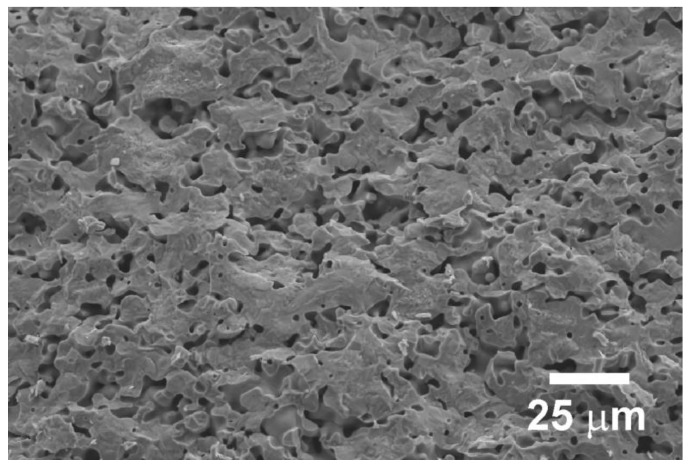
Representative field emission scanning electron microscope (FESEM) micrograph performed on the transversal fractured surface of Ag-free Ca_0.9_Yb_0.1_MnO_3_ samples.

FESEM observations performed on transversal fractured sections of samples showed that no significant differences can be observed among them. The typical microstructure of these samples is illustrated in Figure 2, where a representative image of the Ag-free samples is displayed.

As can be observed, all samples possess well connected grains with relatively high porosity levels. To determine the density of the different samples, Archimedes’ method was used, and the results are presented in Table 1.

These data clearly indicate that Ag addition increases density up to 5 wt.% content, with a subsequent decrease for higher Ag additions. This trend can be explained by the formation of liquid Ag during sintering, which enhances cation mobility at high temperatures and helps to decrease porosity.

**Figure 3:**
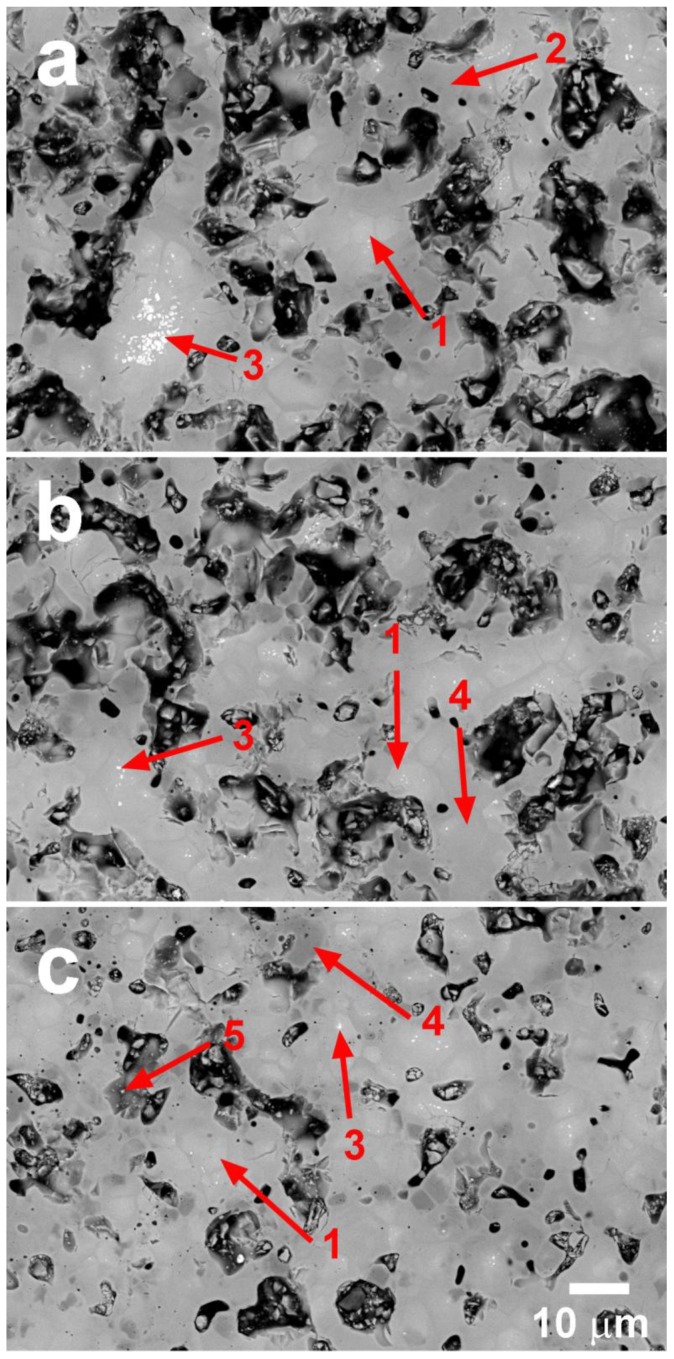
FESEM micrographs performed on representative polished surfaces of Ca_0.9_Yb_0.1_MnO_3_ + x wt.% Ag samples for x = (**a**) 0; (**b**) 1; and (**c**) 5. The different phases are indicated by numbers: #1 is Ca_0.9_Yb_0.1_MnO_3_; #2 is Yb-poor Ca_1−x_Yb_x_MnO_3_; #3 is Yb-rich Ca_1−x_Yb_x_MnO_3_; #4 is the Ca_2_Mn_2_O_5_ secondary phase; and #5 is metallic Ag. Black contrast is associated with porosity.

These aforementioned features can be observed in representative SEM images obtained on polished longitudinal sections of samples. The images are displayed in Figure 3.

In the Ag-free samples, three different phases were identified by their contrast levels via EDS (see Figure 3a). The major sections (grey contrast, #1) correspond to the Ca_0.9_Yb_0.1_MnO_3_ thermoelectric phase, while the dark grey and white sections (#2, and #3) are associated with Yb-poor (Ca_0.5_Yb_0.5_MnO_3_) and Yb-rich (Ca_0.95_Yb_0.05_MnO_3_) compositions, respectively. When Ag was added to samples, modifications to these phases were produced. Yb-rich phase levels drastically decreased, and those of the Yb-poor phase disappeared, confirming the effect of the liquid phase on cation diffusion. In addition, a new Yb-free Ca_2_Mn_2_O_5_ secondary phase (#4) was formed in this process, probably due to a phase equilibrium modification induced by Ag. Moreover, very small metallic Ag particles (#5) can be found between the grains. These findings agree with XRD analysis, as previously discussed. Another characteristic which matches well with the density values displayed in Table 1 is the decrease in porosity with Ag addition up to 5 wt.%, as can be observed in Figure 3 (see black contrast).

**Figure 4:**
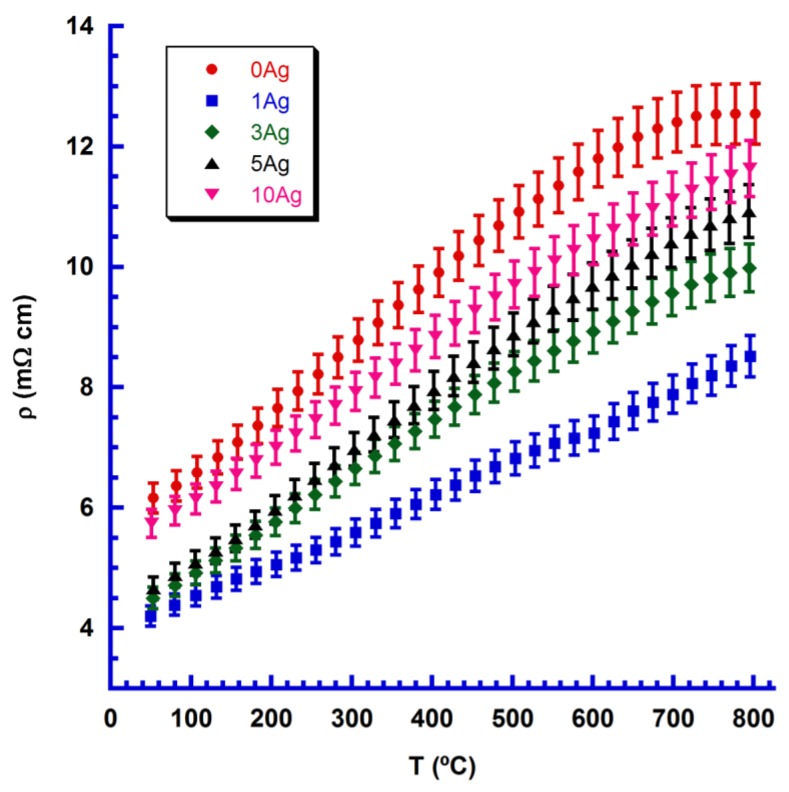
Electrical resistivity change with temperature for Ca_0.9_Yb_0.1_MnO_3_ + x wt.% Ag samples (given as a function of Ag content), together with their measurement errors.

Changes in electrical resistivity with temperature and Ag content are presented in Figure 4, together with their estimated errors (4%), in agreement with previously determined errors for this measuring system [34].

In Figure 4, it is easy to observe that Ag addition leads to a decrease in electrical resistivity, when compared with the Ag-free one, independently of Ag content, for the whole measured temperature range. Moreover, 1 wt.% Ag addition leads to a drastic decrease in electrical resistivity, due to the increase in density, more homogeneous Yb distribution, and a smaller amount of the Ca_2_Mn_2_O_5_ secondary phase. Greater Ag content promotes a slight increase in resistivity, due to higher Ca_2_Mn_2_O_5_ secondary phase levels. It is also clear that Ag does not drastically change sample behavior, which was metallic-like for all samples (dρ/dT > 0) in agreement with previous research [9,14,19,20,21,22]. The lowest value measured for 1 wt.% Ag samples, at room temperature (4 mΩ·cm), is much lower than that reported for undoped CaMnO_3_ (125 mΩ·cm) [35], reflecting the effect of electron doping induced by Yb. These values are in the range reported previously for Yb-doped sintered materials (2.5–7 mΩ·cm) [9,18,19,20], but were obtained via a much shorter sintering process (1.5 h in this study compared with 10–120 h in the literature). At high temperature (800 °C), resistivity values (8 mΩ·cm) follow the same trend; they are lower than that reported for undoped materials (35 mΩ·cm) [35], and are in the range of those for Yb-doped materials (4.2–9 mΩ cm) sintered for longer times [9,18,19,20].

**Figure 5:**
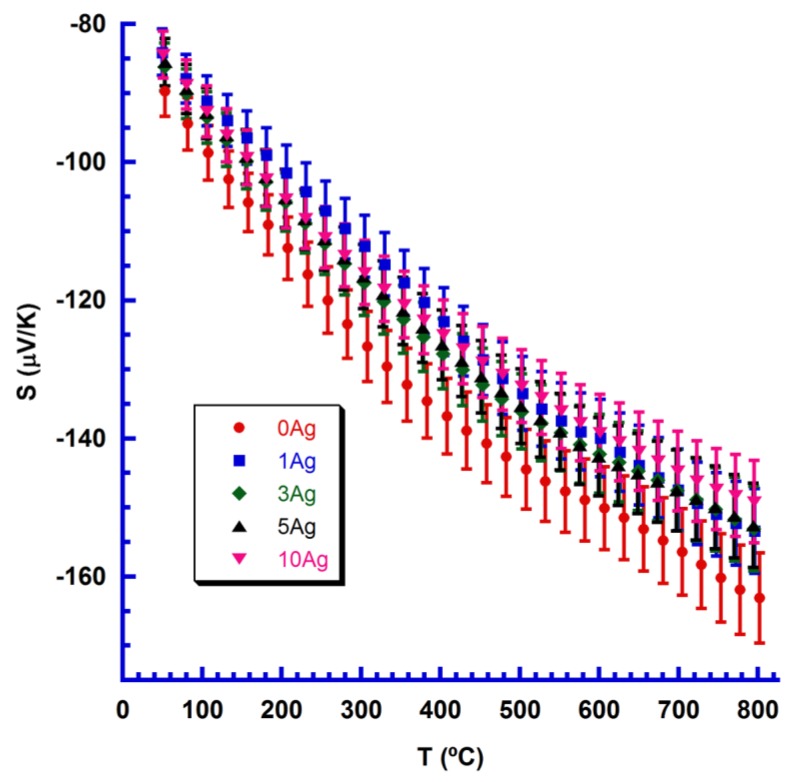
Seebeck coefficient change with temperature for Ca_0.9_Yb_0.1_MnO_3_ + x wt.% Ag samples (given as a function of Ag content), together with their measurement errors.

Figure 5 displays Seebeck coefficient change with temperature for samples with different Ag content, together with their estimated errors (4%), in agreement with previously determined errors for this measuring system [34]. In all samples, S is negative, indicating that the main conduction mechanism is driven by electrons.

Furthermore, absolute S values decreased with Ag addition, compared with Ag-free samples, which is in agreement with their lower electrical resistivity. Consequently, the highest absolute values have been determined as belonging to Ag-free samples across the whole measured temperature range. The highest absolute value at room temperature (90 µV/K) is much lower than that measured for undoped materials (around 350 µV/K) [35] due to higher carrier concentration induced by Yb-doping in CaMnO_3_. This is within the range of that reported for Yb-doped samples (90–100 µV/K) [9,18,19,20]. At high temperature (800 °C) these relationships are maintained, with the measured value (around 160 µV/K) lower than for Yb-free samples (225 µV/K) [35], and about the same as for that measured in Yb-doped samples (140–160 µV/K) [9,18,20].

**Figure 6:**
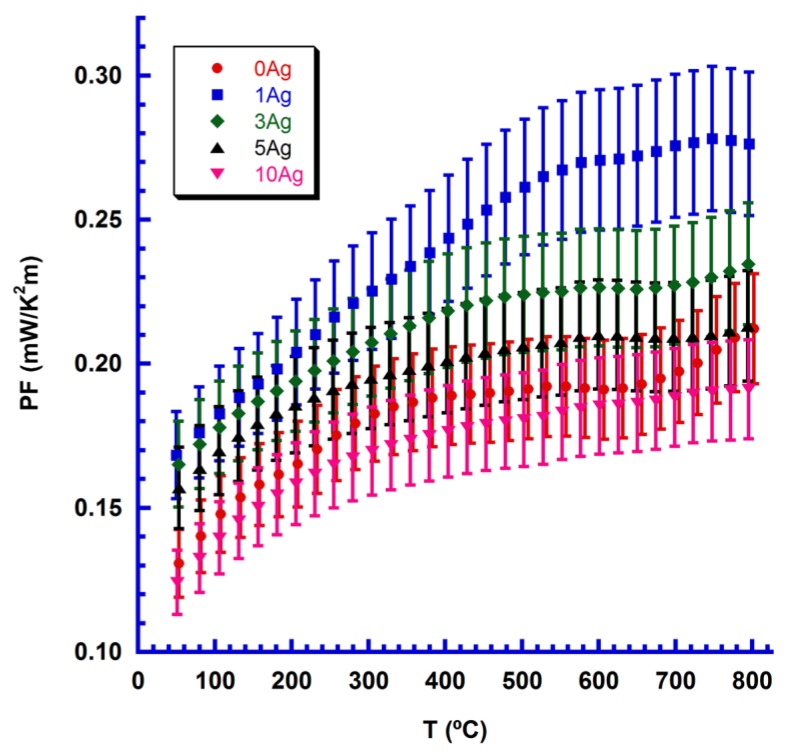
PF change with temperature for Ca_0.9_Yb_0.1_MnO_3_ + x wt.% Ag samples (given as a function of Ag content), together with their measurement errors.

Using these electrical data, sample performances were calculated using their PF values. a PF variation with temperature as a function of Ag content is illustrated in Figure 6, together with estimated errors of around 9%.

The highest PF values were found in 1 wt.% Ag samples, mainly due to a significant decrease in electrical resistivity. The maximum value at room temperature (0.17 mW/K^2^m) is higher than that reported for Yb-free CaMnO_3_ (0.1 mW/K^2^m) [35]. However, it is slightly lower than that determined for Yb-doped materials (0.17–0.23 mW/K^2^m) [9,18,19,20], but these materials were sintered for longer times. Finally, at high temperatures (800 °C) the highest value (0.28 mW/K^2^m) is still much higher than that reported for CaMnO_3_ (0.17 mW/K^2^m) [35], and slightly higher than that obtained for Yb-doped materials (0.22–0.25 mW/K^2^m) [9,18,20].

These data provide evidence that high thermoelectric performance in Yb-doped CaMnO_3_ materials can be achieved via additions of Ag in small amounts. Metallic Ag promotes the formation of a liquid phase during sintering. This phase enhances cation mobility and density of samples within a very short processing time. However, it will still be necessary to optimize thermal treatment in order to decrease the amount of secondary phases before these materials can be used practically in thermoelectric devices. Even if Ag prices seem to be a drawback for their production, though, it should be mentioned that their costs are about 10 times lower than for those associated with the Yb-oxide.

## 4. Conclusions

In this study, Yb-doped CaMnO_3_ thermoelectric ceramics with different metallic Ag additions were successfully prepared via the classical ceramic method. Microstructure observations showed that Ag addition improves Yb cation diffusion and decreases porosity, which was confirmed by density measurements. On the other hand, Ag addition promotes the formation of a Yb-free Ca_2_Mn_2_O_5_ secondary phase. Despite the presence of this secondary phase, electrical resistivity decreased significantly, without a drastic decrease in the absolute Seebeck coefficient. The greatest improvement in thermoelectric performance was observed in 1 wt.% Ag-added samples. These maximum values are slightly higher than the best of those reported for Yb-doped sintered materials, with the advantage of having required a much shorter sintering procedure (1.5 h, compared with 10–120 h in the literature). It can be concluded that this procedure is very advantageous to acquiring the best properties reported in the literature for CaMnO_3_ materials, while decreasing, at the same time, the energy consumption used during the sintering process.

## Figures and Tables

**Table 1:** Mean densities of Ca_0.99_Yb_0.1_MnO_3_ + x wt.% Ag samples, together with their standard errors. Relative densities are also given, taking as the theoretical density 4.99 g/cm^3^ for Ca_0.99_Yb_0.1_MnO_3_ [20], and 10.5 g/cm^3^ for Ag [28].

x	Density (g/cm^3^)	Standard Error	Relative Density (%)
0	3.74	0.05	75
1	4.14	0.03	83
3	4.37	0.04	86
5	4.64	0.02	91
10	4.53	0.03	86
